# Development of a logic regression-based approach for the discovery of host- and niche-informative biomarkers in *Escherichia coli* and their application for microbial source tracking

**DOI:** 10.1128/aem.00227-24

**Published:** 2024-06-28

**Authors:** Daniel Yu, Martin Andersson-Li, Sharon Maes, Lili Andersson-Li, Norman F. Neumann, Monica Odlare, Anders Jonsson

**Affiliations:** 1 School of Public Health, University of Alberta, Edmonton, Alberta, Canada; 2 Aquabiota, Stockholm, Sweden; 3 Department of Natural Sciences, Design and Sustainable Development, Mid Sweden University, Östersund, Sweden; 4 Department of Microbiology, Tumor and Cell Biology, Karolinska Institutet, Solna, Sweden; Centers for Disease Control and Prevention, Atlanta, Georgia, USA

**Keywords:** logic regression, supervised learning, microbial source tracking, source attribution, *Escherichia coli*

## Abstract

Microbial source tracking leverages a wide range of approaches designed to trace the origins of fecal contamination in aquatic environments. Although source tracking methods are typically employed within the laboratory setting, computational techniques can be leveraged to advance microbial source tracking methodology. Herein, we present a logic regression-based supervised learning approach for the discovery of source-informative genetic markers within intergenic regions across the *Escherichia coli* genome that can be used for source tracking. With just single intergenic loci, logic regression was able to identify highly source-specific (i.e., exceeding 97.00%) biomarkers for a wide range of host and niche sources, with sensitivities reaching as high as 30.00%–50.00% for certain source categories, including pig, sheep, mouse, and wastewater, depending on the specific intergenic locus analyzed. Restricting the source range to reflect the most prominent zoonotic sources of *E. coli* transmission (i.e., bovine, chicken, human, and pig) allowed for the generation of informative biomarkers for all host categories, with specificities of at least 90.00% and sensitivities between 12.50% and 70.00%, using the sequence data from key intergenic regions, including *emrKY–evgAS*, *ibsB–(mdtABCD-baeSR), ompC–rcsDB,* and *yedS–yedR*, that appear to be involved in antibiotic resistance. Remarkably, we were able to use this approach to classify 48 out of 113 river water *E. coli* isolates collected in Northwestern Sweden as either beaver, human, or reindeer in origin with a high degree of consensus—thus highlighting the potential of logic regression modeling as a novel approach for augmenting current source tracking efforts.

**IMPORTANCE:**

The presence of microbial contaminants, particularly from fecal sources, within water poses a serious risk to public health. The health and economic burden of waterborne pathogens can be substantial—as such, the ability to detect and identify the sources of fecal contamination in environmental waters is crucial for the control of waterborne diseases. This can be accomplished through microbial source tracking, which involves the use of various laboratory techniques to trace the origins of microbial pollution in the environment. Building on current source tracking methodology, we describe a novel workflow that uses logic regression, a supervised machine learning method, to discover genetic markers in *Escherichia coli*, a common fecal indicator bacterium, that can be used for source tracking efforts. Importantly, our research provides an example of how the rise in prominence of machine learning algorithms can be applied to improve upon current microbial source tracking methodology.

## INTRODUCTION

Poor microbiological quality of drinking, recreational, and agricultural water places a significant burden on public health and can lead to substantial economic impacts. As such, the ability to reliably detect and identify the sources of microbial pollution in water, particularly from fecal sources, is paramount for evaluating the risks associated with exposure to contaminated water and the subsequent control of waterborne diseases. One possible strategy involves the use of microbial source tracking tools (i.e., methods focused on detecting fecal microbes that are specifically found in certain animal hosts) to trace the origin and sources of fecal contamination in aquatic environments (1, 2). The practice of microbial source tracking hinges on the assumption that, over time, different subgroups of microorganisms become better adapted to a particular host environment, and by outcompeting the conspecific and allospecific microflora they become the dominant members of the gut microbiome of a given host species. The close association between these microbes and their host then leads to the acquisition of identifiable attributes (e.g., genes, DNA sequence polymorphisms, phenotypes, etc.) that can serve as markers of fecal contamination from that host species (1). Broadly, microbial source tracking approaches can be categorized as either library-dependent or library-independent. Library-dependent approaches involve the characterization of a collection of bacterial isolates derived from known sources to construct a reference library for some phenotypic or genotypic trait, such as antibiotic resistance profiles, serotypes, ribotypes, or DNA fingerprints (1), against which bacterial isolates of unknown origin can be compared with determine their source. In contrast, library-independent approaches typically focus on the direct detection and quantification of host-specific genetic markers to classify samples based on whether they contain fecal contamination from a particular host source (3).

Various library-dependent and library-independent approaches have been developed over the years, contributing to a growing “toolbox” of microbial source tracking methods. Although these methods have been largely employed within the laboratory setting, the relatively recent application of an ever-growing suite of computational techniques for microbiological research (4–7) presents additional opportunities for advancing microbial source tracking methodology. In particular, the use of machine learning could be leveraged for the purposes of discovering novel, host-informative markers for source tracking efforts. Briefly, machine learning involves the use of algorithms to recognize the underlying patterns in large volumes of data (5). These algorithms typically fall under one of two categories: unsupervised learning and supervised learning. Unsupervised machine learning methods, such as k-means clustering, hierarchical clustering, and various dimensionality reduction procedures, are exploratory in nature and do not involve the use of training data or result in a defined target or output (5, 8). As such, unsupervised methods mainly seek to uncover clusters in data without consideration for pre-existing labels that the data may have (i.e., host source). In contrast, supervised machine learning approaches, which include clustering and regression algorithms such as logistic regression, support vector machine, random forest, and neural networks, are first “trained” on an initial set of data such that they can then make future predictions or classifications with new data (5). Supervised methods, therefore, attempt to uncover patterns in data correlating specifically with observed data labels (i.e., host source), making them more appropriate for identifying potential host-informative markers for microbial source tracking.

Machine learning has shown promise for application in various microbiology research areas, including microbial ecology and microbiomes (4, 9–11), antibiotic resistance (12, 13), epidemiology and clinical diagnostics (5, 14–16), and drug discovery (17–20). To a lesser extent, the utility of machine learning has also been evaluated for microbial source tracking purposes. Wu et al. (21), for instance, examined the use of six supervised machine learning algorithms, including K-nearest neighbors, Naïve Bayes, support vector machine, simple neural network, random forest, and XGBoost, to model and predict the major sources of fecal contamination in watersheds. According to ecological factors such as land cover, weather (i.e., temperature), and hydrologic variables (i.e., precipitation), the machine learning algorithms were able to achieve classification accuracies ranging from 69% to 92% when distinguishing between human and non-human sources of fecal contamination.

Modeling sources of fecal contamination based on ecological factors alone, however, does not necessarily inform the development of assayable markers for source tracking efforts. Alternatively, machine learning could be leveraged for the analysis of genomic sequence data to identify key genetic features that can be used to predict the host source of bacterial isolates. Zhang et al. (22), for example, used a random forest approach to identify 50 key genetic determinants that could reliably predict the original livestock source of outbreak-associated *Salmonella* Typhimurium isolates. From the host-informative markers identified, which included 10 core genome single nucleotide polymorphisms (SNPs) and 40 accessory genes related to virulence, metal resistance, and colonization, the authors were able to correctly identify the original source of 7 of 8 major *Salmonella* zoonotic outbreaks in the United States from 1998 to 2013. Opting for a different machine learning algorithm, Lupolova et al. (23) similarly developed support vector machine classifiers to predict the host-specificity of *Salmonella enterica* and *Escherichia coli* isolates. According to the characteristic presence and absence of predicted protein variants, the authors were able to correctly classify the host source of 67%–90% of *S. enterica* isolates collected from avian, bovine, human, and swine hosts, as well as 83% of human and bovine-derived *E. coli* O157 strains. As a follow-up, Lupolova et al. (8) also developed classifiers using random forest and neural network algorithms, which were found to correctly classify roughly 80% of *S*. Typhimurium isolates collected from avian, bovine, human, and swine hosts.

As such, the utility of supervised learning methods for identifying microbial genetic features of host-specificity could be leveraged for the discovery of biomarkers as novel source tracking targets. Despite this, supervised learning approaches such as support vector machines, random forests, and neural networks are often considered “black box” algorithms as they require specialist knowledge to perform and typically generate highly complex results that are difficult to interpret (8). Furthermore, when used for biomarker discovery purposes, these methods often generate large numbers of potential genetic features that could possibly, but not necessarily, reflect host-specificity, making it difficult to pinpoint the specific determinants that can act as reliable biomarkers for source tracking. Instead, alternative supervised learning approaches may be better suited for the discovery of novel source tracking biomarkers. Reflecting this, using a novel logic regression-based modeling approach (24), Zhi et al. (25) identified highly host-specific, biologically plausible SNP-SNP interactions encoded within the *uspC–flhDC, asnS–ompF,* and *csgBAC–csgDEFG* intergenic regions (ITGRs) of the *E. coli* genome. Depending on the host species, anywhere between 31% and 94% of *E. coli* strains collected from a specific host harbored an SNP-SNP biomarker that was at least 96.00% specific to that host. The high specificity of the identified host-informative biomarkers was even observed between closely related host species, such as coyotes and dogs, demonstrating the ability of logic regression to distinguish and classify *E. coli* isolates recovered from taxonomically related host sources. The strength of this approach for biomarker discovery was further demonstrated in a follow-up study by Zhi et al. (26), where logic regression was used to survey the *E. coli* genome for additional host-informative ITGRs that could discriminate between human and bovine-derived isolates. Depending on the ITGRs analyzed, up to 72.00% of human *E. coli* isolates carried a biomarker that was 98.00% specific to human strains, whereas up to 92% of cattle isolates carried a biomarker that was 98.00% specific to cattle strains, suggesting a high degree of host-specificity in the *E. coli* populations colonizing these hosts. Interestingly, the specific ITGRs that were particularly host-informative differed between the two host groups, as the human-specific biomarkers were predominant in ITGRs associated with antibiotic resistance, whereas the bovine-specific biomarkers were mainly found in ITGRs involved in stress regulation.

As logic regression appears to be capable of not only identifying host-informative genetic regions in microbial genomes but also generating biologically plausible biomarkers of host-specificity, it may be well-suited for the discovery of novel genetic markers for source tracking. Building on the workflows originally laid out by Zhi et al. (25, 26), we describe a novel logic regression-based biomarker discovery method to identify host-informative SNP biomarkers within ITGRs across the *E. coli* genome. In our case, *E. coli* was chosen as a suitable target for the discovery of source-informative biomarkers for source tracking as it already serves as one of the most important indicator microorganisms currently under surveillance for microbial water quality assessment purposes. Using a whole genome-based, *in silico* approach, we first demonstrate the utility of logic regression for identifying highly host-specific biomarkers within *E. coli* strains recovered from a wide range of host and niche sources. Adapting our methodology for practical use, we then generate human-, reindeer- and beaver-specific biomarkers to classify *E. coli* isolates recovered via water samples taken from the Indalsälven river in Northwestern Sweden (27)—thus highlighting the potential of logic regression modeling as a novel source tracking approach.

## RESULTS

### Construction of local *E. coli* genome repository and ITGR candidate list for *in silico* biomarker discovery

**Fig 1:**
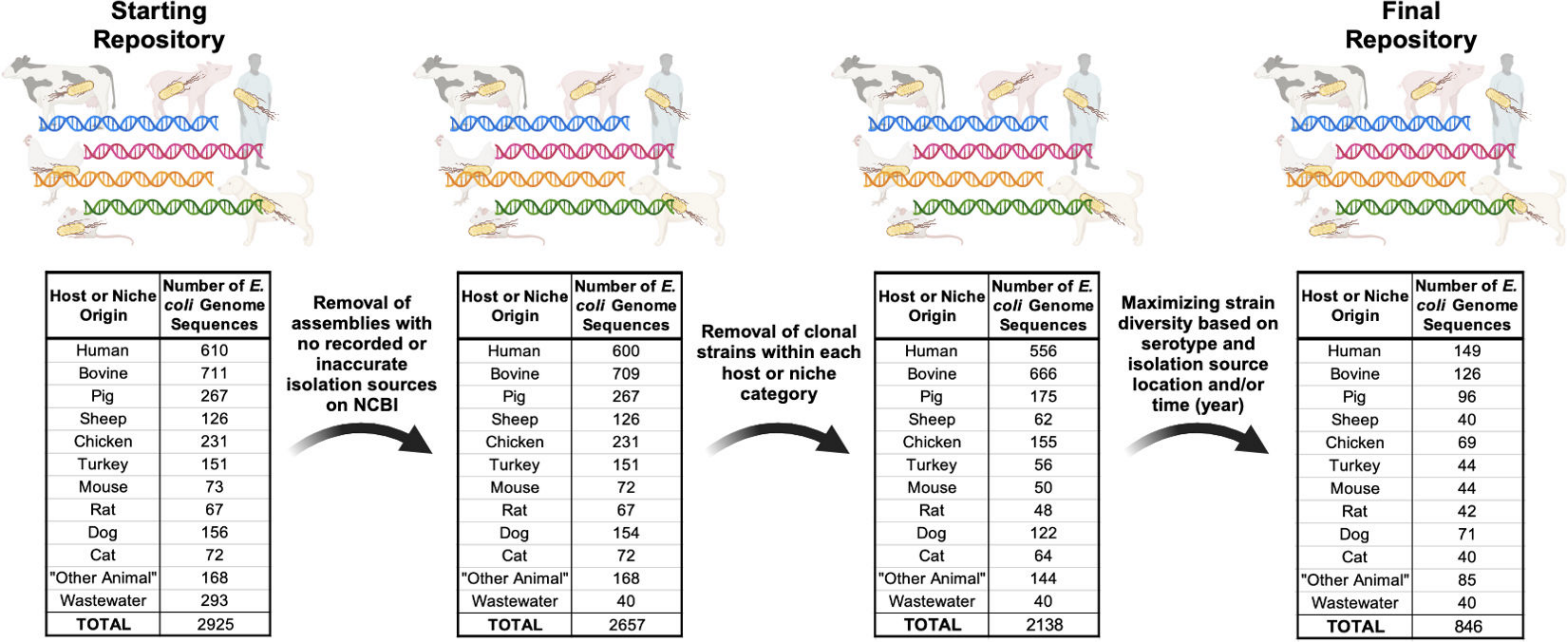
Flowchart depicting construction and refinement of local *E. coli* genome sequence repository for the in-silico logic regression analyses. The genome sequences of 2925 *E. coli* strains isolated from a wide range of host (i.e., human, bovine, pig, sheep, chicken, turkey, mouse, rat, dog, cat, and other animal hosts with lower representation in the repository) and niche (i.e., wastewater) sources were downloaded from NCBI. The repository was then refined through (i) the removal of assemblies with inaccurate or no available isolation source information; (ii) the removal of clonal strains to reduce the degree of clonal representation in the repository; and (iii) maximizing strain diversity through representation of serotypes and isolation source location/time. The final 846 “representative” strains across the host species and niche source categories comprised the final repository, which was then used for the downstream in-silico logic regression analyses.

A total of 2925 *E. coli* genome sequences were downloaded from NCBI to construct a local genome repository for the *in silico* logic regression analyses, including 610 human strains, 711 bovine strains, 267 pig strains, 126 sheep strains, 231 chicken strains, 151 turkey strains, 73 mouse strains, 67 rat strains, 156 dog strains, 72 cat strains, 168 strains that were grouped into an “other animal” category due to the limited representation of their host source in the repository, and 294 wastewater strains. The initial repository was then screened to maximize sequence quality and strain diversity (i.e., limiting clonal representation), resulting in a final repository of “representative” *E. coli* genome sequences from each host and niche source category (Fig. 1). After screening, the final repository consisted of 846 *E. coli* genome sequences including 149 human strains, 126 bovine strains, 96 pig strains, 40 sheep strains, 69 chicken strains, 44 turkey strains, 44 mouse strains, 42 rat strains, 71 dog strains, 40 cat strains, 85 strains from other animals as a negative control group for the other host categories, and 40 wastewater strains as an additional non-host associated, negative control group (Table S1).

Expanding on a set of ITGRs that were previously found to be host-informative (25, 26), including the *flhDC–uspC*, *asnS–ompF*, *csgDEFG–csgBAC*, and *yedS–yedR* loci, 63 total candidate ITGRs were evaluated for biomarker discovery purposes (Table S2). ITGRs were selected based on the role of the flanking genes in functions that could be relevant for survival within, and subsequent colonization of, a given host species’ gastrointestinal environment, including adhesion and colonization factors, including various fimbrial and pili systems; stress resistance, including heat shock, acid stress, antibiotic stress, and stress responses mediating environmental persistence during transmission between hosts; motility and flagellar systems; and nutrition, including metabolic pathways for various sugar substrates. Of the 62 candidate ITGRs identified, 29 were found to be sufficiently represented across the strains in the repository (i.e., in at least 750 strains), of satisfactory length (i.e., at least 250 bp), and displayed sufficient sequence diversity and were thus retained for analysis with logic regression.

### Single-ITGR logic regression-based biomarker discovery analysis with expanded source range

Logic regression was used to generate source-specific logic models, representing source-informative SNP-SNP biomarkers, based on the sequence variation contained within each of the 29 candidate ITGRs for each host- and niche-source represented in the expanded repository. Following previous studies (25, 26), the performance of the generated logic models was evaluated according to two parameters: (i) sensitivity, which was defined as the proportion of strains derived from a target source category that carried the corresponding source-specific SNP pattern; and (ii) specificity, which was defined as the proportion of strains from sources other than the target source category (i.e., all other host or niche sources) that did not carry the source-specific SNP biomarker of interest. Although previous work using logic regression for biomarker discovery purposes restricted the model building parameters to just 2 trees and 10 leaves (25, 26), the iterative approach utilized in this study revealed that the “best” model size differed based on the source category and ITGR sequence analyzed. Depending on the specific source-ITGR pairing, the generated logic models varied in size, ranging from as small as 3 trees and 16 leaves to as large as 3 trees and 25 leaves (Table S4). Despite the iterative approach to model building, however, not all ITGRs were found to be source-informative as several single-ITGR logic models produced for each source category were found to be 0% sensitive, indicating that the biomarkers produced were not found in any of the strains constituting the given source category. Regardless, source-informative logic models could still be generated for each source category, though the performance of the models varied depending on the specific host- or niche-source and the specific ITGR locus analyzed.

Of the logic models generated that were source-informative to some degree (i.e., with greater than 0% sensitivity), sensitivities ranged from as low as 3.13% to as high as 66.67% while specificities ranged between 84.25% and 100.00% (Table S4). In line with previous studies (26), the degree of source-related information carried within single ITGRs appeared to vary depending on the specific host- or niche-source. Indeed, the most informative ITGRs differed across each host- and niche-source (Table 1), indicating that there was no single ITGR that was generally informative across all host and niche-source categories represented in the repository. Furthermore, while logic models could be generated for each source category, the biomarker discovery approach appeared to be especially effective for certain source-categories. Reflecting this, logic models of at least 30.00% sensitivity and over 97.00% specificity were produced for the pig (i.e., in the *csgDEFG–csgBAC* locus), sheep (i.e., in the *flhDC–uspC* and *yjjP–[yjjQ-bglJ]* loci), and mouse (i.e., in the *ompC–rcsDB,* and *nanCMS–fimB* loci) groups, with select mouse-informative biomarkers exceeding 50.00% sensitivity and 97.00% specificity (i.e., in the *yedS–yedR* and *csgDEFG–csgBAC* loci). Interestingly, although the wastewater group served as a non-host associated negative control for the other host-categories, the biomarkers generated for the wastewater strains were among the best performing with sensitivities ranging from 37.50% to 50.00% and specificities exceeding 99.00% (Table 1).

**TABLE 1:** Top five performing intergenic regions for each host/niche-category in the expanded repository, as determined via logic regression with 10-fold cross-validation

Top 5 ITGRs	Bovine		Top 5 ITGRs	Cat		Top 5 ITGRs	Chicken	
	Sensitivity	Specificity		Sensitivity	Specificity		Sensitivity	Specificity
*azuC*	13.64%	100%	*csgDEFG*	16.67%	99.34%	*araC*	12.50%	100%
*gadE*	9.09%	100%	*flhDC*	11.11%	98.66%	*mpC*	25.00%	99.38%
*ykgR*	9.09%	100%	*mdtABCD*	14.29%	98.75%	*bdm*	11.11%	99.36%
*fimE*	10.00%	97.64%	*acrEF*	16.67%	96.13%	*bssS*	9.09%	100%
*yedS*	31.25%	84.25%	-	-	-	*bluF*	11.11%	98.68%
**Top 5 ITGRs**	**Dog**		**Top 5 ITGRs**	**Human**		**Top 5 ITGRs**	**Mouse**	
	**Sensitivity**	**Specificity**		**Sensitivity**	**Specificity**		**Sensitivity**	**Specificity**
*mpF*	12.50%	98.65%	*ftnB*	17.65%	99.33%	*mpC*	37.50%	100%
*emrKY*	5.56%	100%	*gadE*	8.70%	99.31%	*rpoE*	14.29%	100%
*bdm*	5.56%	100%	*fucPIKUR*	12.50%	97.79%	*fimB*	33.33%	100%
*yobF*	5.56%	99.33%	*ecpRABCDE*	21.74%	93.94%	*yedS*	62.50%	97.78%
*mdtABCD*	9.09%	100%	*flhDC*	20.69%	92.25%	*csgDEFG*	66.67%	97.26%
**Top 5 ITGRs**	**Pig**		**Top 5 ITGRs**	**Rat**		**Top 5 ITGRs**	**Sheep**	
	**Sensitivity**	**Specificity**		**Sensitivity**	**Specificity**		**Sensitivity**	**Specificity**
*csgDEFG*	20.00%	97.97%	*mpF*	22.22%	100%	*flhDC*	33.33%	100%
*yobF*	12.50%	98.68%	*fimE*	18.18%	100%	*ftnB*	20.00%	100%
*rpoE*	30.00%	97.99%	*gadE*	16.67%	100%	*acrEF*	14.29%	100%
*rcsA*	19.05%	99.31%	*ypfM*	12.50%	100%	*yehABCD*	14.29%	100%
*agaR*	11.11%	99.32%	*csgDEFG*	12.50%	100%	*yjjQ*	33.33%	98.08%
**Top 5 ITGRs**	**Turkey**		**Top 5 ITGRs**	**Wastewater**				
	**Sensitivity**	**Specificity**		**Sensitivity**	**Specificity**			
*ypfM*	16.67%	99.38%	*ftnB*	50.00%	100%			
*csgDEFG*	25.00%	98.05%	*araC*	37.50%	100%			
*fimB*	28.57%	98.62%	*fimB*	37.50%	100%			
*fucPIKUR*	14.29%	99.35%	*azuC*	44.44%	100%			
*mpC*	11.11%	100%	*ypfM*	44.44%	99.36%			

### Logic regression-based biomarker discovery analysis with reduced source range

Although source-informative biomarkers of varying degrees of sensitivity and specificity could be generated for each source category in the expanded repository, the variability observed in the performance of the biomarkers and in the specific loci from which they were generated limit their applicability and scalability for use in practical source tracking assays. Ideally, for the generated biomarkers to be useful for source tracking efforts, they would need to be produced from the same input sequence and should be informative across a range of host- and/or niche-sources simultaneously. To address these concerns, a second logic regression analysis was performed to identify source-informative SNP biomarkers, but with two modifications: first, the host range was reduced to include only bovine, chicken, human, and pig strains, thereby reflecting the major zoonotic and food-associated routes for *E. coli* transmission and providing a practical application for the biomarker discovery process; and second, concatenated ITGR sequences were used as input for logic regression to identify target sequences that were informative across all host groups surveyed, as well as to improve the sensitivity and specificity of the generated biomarkers (25, 26).

As in the first biomarker discovery analysis with the expanded source range, a significant degree of variability was observed across the single-ITGR-based logic models that were generated for each host category. The “optimal” model size differed depending on the host source of interest and the specific ITGR analyzed, though to a lesser extent when compared with the logic models generated with the expanded source repository, as the generated biomarkers ranged in size from 3 trees and 20 leaves to 3 trees and 24 leaves (Table S5). Similarly, the performance of the generated biomarkers also varied across host categories and ITGRs. Although there were many single-ITGR logic models with 0% sensitivity, biomarkers with sensitivities between 3.85% and 76.92% and specificities ranging from 65.00% to 100.00% were still generated across the host categories represented in the reduced repository. Interestingly, with the reduced host range the performance of the logic models appeared to improve for select host categories. The human-informative logic models, for instance, improved drastically during the biomarker discovery analysis with a reduced host range, with sensitivities reaching as high as 61.29% and specificities exceeding 94.00% (Table 2). Though to a lesser extent, the bovine-informative models also improved with the bovine biomarkers displaying sensitivities as high as 26.92% and specificities of at least 91.00%. In contrast, when compared with the original biomarker discovery analysis with the expanded source repository, the generated logic models for the chicken and pig groups exhibited similar sensitivities and specificities, with no clear improvement in model performance between the two analyses.

**TABLE 2:** Top five performing intergenic regions for each host-category in the reduced repository, as determined via logic regression with 10-fold cross-validation

Top 5 ITGRs	Bovine		Top 5 ITGRs	Chicken		Top 5 ITGRs	Human		Top 5 ITGRs	Pig	
	Sensitivity	Specificity		Sensitivity	Specificity		Sensitivity	Specificity		Sensitivity	Specificity
*ecpRABCDE*	18.18%	98.33%	*azuC*	16.67%	100.00%	*hns*	61.29%	94.74%	*rpoE*	28.57%	95.95%
*acrEF*	24.00%	95.00%	*mpC*	16.67%	96.05%	*yedS*	43.48%	96.15%	*mpC*	21.05%	100.00%
*emrKY*	26.92%	91.94%	*mdtABCD*	10.00%	100.00%	*emrKY*	38.71%	94.74%	*yobF*	25.00%	91.55%
*ftnB*	23.08%	93.55%	*fimE*	11.11%	98.53%	*mdtABCD*	33.33%	94.44%	*yjjQ*	22.22%	92.75%
*yobF*	23.08%	91.80%	*yedS*	40.00%	87.69%	*acrEF*	67.74%	88.89%	*fimB*	15.00%	100.00%

With the significant variability observed across the single-ITGR logic models, no single ITGR seemed to be adequately informative across each of the host categories represented in the reduced repository. As such, to identify an input target sequence that could be used to generate host-informative biomarkers for each host group, candidate ITGR sequences were concatenated and re-analyzed using logic regression. Specifically, ITGRs that were found to produce informative biomarkers for more than one host group (i.e., *emrKY–evgAS*, *yedS–yedR*, *ompC–rcsDB*, and *ibsB–[mdtABCD-baeSR]*) were chosen for concatenation. Upon re-analysis, three concatenated sequences were found to be informative, to varying degrees, across multiple host groups. The *emrKY–yedS–mdtABCD* locus appeared to be the most host informative, as the generated biomarkers were found to be significantly associated with each host category (*P* < 0.05), with sensitivities exceeding 30.00% and specificities of at least 92.00%—though their classification accuracies ranged from as low as 75.34% for the bovine model to as high as 83.56% each for the chicken and pig models (Table 3). To a lesser extent, the concatenated sequence *yedS–ompC–emrKY* was also found to be moderately informative across all host groups surveyed, with sensitivities of at least 25.00% and specificities of at least 91.00%; however, while the bovine, chicken, and human models were found to be significantly associated with their corresponding host group (*P* < 0.01), the pig biomarker did not appear to be significantly correlated with the pig strains (*P* = 0.216). Furthermore, the classification accuracies of these biomarkers were found to vary widely depending on the host source, as they ranged from as low as 70.27% for the bovine biomarker and as high as 86.49% for the chicken biomarker. Conversely, while the concatenated sequence *emrKY–ompC–mdtABCD* was found to be less informative for the chicken and pig groups, the generated bovine models (sensitivity of 46.15% and specificity of 96.67%) and human models (sensitivity of 70.00% and specificity of 91.07%) were the best performing across all three concatenated target sequences and the most significantly associated with their respective host categories (*P* < 0.001), suggesting that this target sequence could be particularly useful for identifying *E. coli* strains originating from bovine and/or human hosts. Interestingly, despite being particularly informative specifically for the bovine and human groups, all biomarkers generated from the *emrKY–ompC–mdtABCD* target appeared to be most effective for classification purposes as all host-associated biomarkers exhibited classification accuracies exceeding 80.00%.

**TABLE 3:** Performance and strength of association of generated logic models with each host category, as determined with logic regression analysis on concatenated ITGR sequences and 10-fold cross validation

Concatenated ITGR sequence	Host category	Model size	Model	Sensitivity	Specificity	Accuracy		*P* value (ANOVA)
emrKY-yedS- mdtABCD	Bovine	3 trees, 21 leaves	=−19.5 + 19.9 * (((yedS375_C and yedS302_T) and (not emrKY210_A)) and ((mdtABCD68_T or (not yedS242_A)) and (yedS325_T and (not yedS290_T)))) +2.08 * (((emrKY326_G and (not mdtABCD54_G)) or ((not yedS300_A) or mdtABCD130_A)) and (((not emrKY6_A) and mdtABCD160_T) and (not yedS256_T))) −2.77 * ((((not yedS237_T) or (not mdtABCD108_A)) or (yedS24_A and emrKY131_A)) or ((not emrKY6_A) or (emrKY6_A and (not emrKY6_A))))	39.13%	92.00%	75.34%		<0.001
	Chicken	3 trees, 24 leaves	=-5.45–20 * (((emrKY394_G or (not mdtABCD94_C)) and (yedS171_G or (not mdtABCD96_C))) and (((not yedS318_T) or yedS383_A) or (yedS135_C or yedS379_C))) +2.8 * (((emrKY196_C or yedS237_C) or ((not yedS345_C) or yedS351_A)) or (((not mdtABCD184_A) and yedS37_A) or (yedS243_T and (not yedS129_T)))) +3.9 * ((((not yedS88_A) and yedS351_G) and ((not yedS237_C) and yedS24_A)) or ((yedS211_A and yedS262_A) or (emrKY197_A and yedS317_C)))	35.71%	94.92%	83.56%		0.004
	Human	3 trees, 22 leaves	=-0.388–2.86 * ((((not mdtABCD184_C) or emrKY359_C) or ((not emrKY248_C) or (not yedS37_C))) or (((not yedS87_T) or mdtABCD19_A) or mdtABCD43_T)) +2.87 * (((yedS375_T or emrKY135_G) or (mdtABCD74_C and (not yedS274_C))) or (((not yedS242_G) and mdtABCD108_A) or (yedS325_C or yedS280_-))) +4.28 * (((emrKY238_A or (not yedS80_A)) or (not yedS426_G)) or ((yedS87_C or (not yedS377_T)) or (emrKY59_C or mdtABCD46_A)))	50.00%	92.45%	80.82%		0.003
	Pig	3 trees, 24 leaves	=−7.58 + 3.79 * (((yedS49_T and (not mdtABCD54_A)) and (mdtABCD148_C and yedS274_T)) or (((not emrKY173_A) or (not emrKY173_A9)) or (yedS270_T or (not yedS492_G)))) +6.28 * (((emrKY218_G and mdtABCD68_T) or (emrKY203_C or yedS18_T)) or (((not yedS371_G) or (not yedS378_G)) or (yedS185_T or yedS287_T))) +5.54 * ((((not emrKY50_T) or mdtABCD184_A) and ((not yedS80_T) and (not mdtABCD19_A))) and ((yedS383_C and yedS107_T) and ((not emrKY238_A) and (not yedS281_C))))	31.25%	94.74%	83.56%		0.011
emrKY-ompC- mdtABCD	Bovine	3 trees, 24 leaves	=0.619 + 5.14 * ((((not emrKY394_A) and rcsDB86_A) and (emrKY131_A and rcsDB159_A)) and ((emrKY326_A or (not mdtABCD130_C)) or (emrKY108_G or (not ompC230_C)))) −4.92 * (((mdtABCD54_G and (not rcsDB212_A)) or ((not rcsDB129_T) or (not emrKY247_C))) and (((not ompC102_-) and (not rcsDB55_T)) and ((not emrKY398_T) and (not mdtABCD39_T)))) −1.73 * ((((not emrKY242_T) and ompC231_C) or ((not rcsDB39_C) or emrKY280_A)) and ((mdtABCD1_A and (not rcsDB134_A)) and (rcsDB56_T and ompC219_C)))	46.15%	96.67%		81.40%	<0.001
	Chicken	3 trees, 24 leaves	=3.3–6.31 * (((emrKY210_G and rcsDB87_G) and ((not rcsDB210_A) or mdtABCD160_G)) and (((not mdtABCD28_G) and (not emrKY6_A)) and ((not ompC88_T) and emrKY6_A))) +5.14 * ((((not rcsDB210_T) and (not ompC146_A)) and (emrKY6_A and (not emrKY6_A))) and ((mdtABCD94_A and (not rcsDB107_T)) or (emrKY16_T and (not ompC184_T)))) −3.89 * (((rcsDB179_A and (not ompC233_T)) and (emrKY6_A86 or (not ompC247_T))) and (((not emrKY196_C) and (not mdtABCD72_A)) and (emrKY6_ and emrKY6_A)))	21.43%	98.61%		86.05%	0.008
	Human	3 trees, 23 leaves	=5.77–4.17 * (((emrKY394_G and (not mdtABCD144_T)) and ((not rcsDB69_T) and emrKY280_G)) or (emrKY398_T or ((not mdtABCD32_G) or (not rcsDB179_A)))) −5.26 * ((((not emrKY92_A) and ompC185_G) or (mdtABCD19_A or mdtABCD168_G)) or (((not ompC174_A) or (not rcsDB55_C)) or ((not ompC88_C) or (not rcsDB61_A)))) −3.11 * ((((not emrKY217_T) or (not mdtABCD54_G)) or ((not ompC33_T) or ompC161_C)) and ((rcsDB24_T and (not ompC271_A)) and ((not emrKY247_T) and (not rcsDB21_T))))	70.00%	91.07%		83.72%	<0.001
	Pig	3 trees, 23 leaves	=4.29–1.97 * ((((not emrKY50_C) or ompC334_T) and (mdtABCD219_T and emrKY173_A)) or ((mdtABCD92_T or rcsDB188_-) and (ompC157_G and (not emrKY173_A8)))) −20.4 * ((((not emrKY394_G) or ompC257_C) and ((not mdtABCD43_T) or (not ompC337_T))) or (((not emrKY92_A) or ompC322_A) and ompC185_C)) −4.37 * (((rcsDB129_T and ompC157_G) and ((not emrKY184_A) and (not mdtABCD187_A))) and ((rcsDB276_T and (not ompC21_A)) and ((not emrKY361_T) and (not rcsDB61_-))))	12.50%	100.00%		83.72%	0.007
yedS-ompC- emrKY	Bovine	3 trees, 22 leaves	=0.0145–4.24 * (((yedS429_- or yedS236_C) and (rcsDB56_T or (not ompC334_A))) and (((not emrKY248_T) and (not yedS358_C)) and ((not emrKY37_C) and (not yedS217_C)))) −18.7 * (((not emrKY135_A) or (not emrKY248_T)) and (((not yedS150_C) or (not yedS302_T)) or (yedS375_T or (not yedS74_G)))) +3.46 * ((((not emrKY359_T) or (not yedS242_A)) and ((not rcsDB129_C) and (not yedS325_C))) and ((rcsDB30_T or (not rcsDB302_C)) or (yedS429_- or yedS317_A)))	30.77%	91.67%		70.27%	0.007
	Chicken	3 trees, 20 leaves	=1.96–20.8 * (((emrKY217_T or (not ompC80_A)) or (yedS273_A or rcsDB21_T)) and (ompC88_C and ((not ompC220_C) or yedS211_A))) −3.43 * ((not yedS384_T) or (((not rcsDB123_A) or (not yedS269_A)) and ((not ompC257_C) and (not yedS37_A)))) −3.42 * (((ompC337_T or yedS202_T) or (yedS379_C or (not rcsDB257_T))) or (((not rcsDB63_C) or (not yedS158_A)) or ((not yedS80_A) or ompC220_T)))	30.00%	95.31%		86.49%	0.004
	Human	3 trees, 20 leaves	=-1.4–4.79 * ((yedS114_A or (not rcsDB233_A)) or ((not rcsDB188_-) and ((not yedS318_T) or yedS217_C))) +6.42 * ((((not ompC247_T) and (not yedS140_T)) or (rcsDB56_C or yedS384_C)) and (((not yedS236_G) and yedS419_C) and ((not emrKY217_C) or ompC231_G))) +5.19 * ((((not yedS150_C) or (not yedS377_T)) or emrKY218_A) or ((emrKY125_A or (not ompC272_T)) or (yedS76_T or (not emrKY350_A))))	36.36%	94.23%		77.03%	0.009
	Pig	3 trees, 24 leaves	=−18.3 + 22.9 * ((((not yedS90_A) and (not ompC184_T)) and (yedS241_C and (not ompC258_G))) or ((emrKY197_A and (not yedS375_T)) and (yedS197_A and rcsDB154_A))) −5.44 * ((((not yedS287_T) and yedS492_G) and (emrKY173_A and (not emrKY203_C))) and (((not yedS90_A) and yedS288_A) and (yedS241_C and (not yedS185_T)))) −3.46 * ((((not emrKY273_T) or (not ompC231_C)) or (yedS317_A or (not ompC334_A))) and ((yedS316_T or yedS87_C) or ((not ompC334_T) or (not yedS156_T))))	25.00%	93.10%		78.38%	0.216

### Application of logic regression for source attribution of environmental water *E. coli* isolates

**Fig 2:**
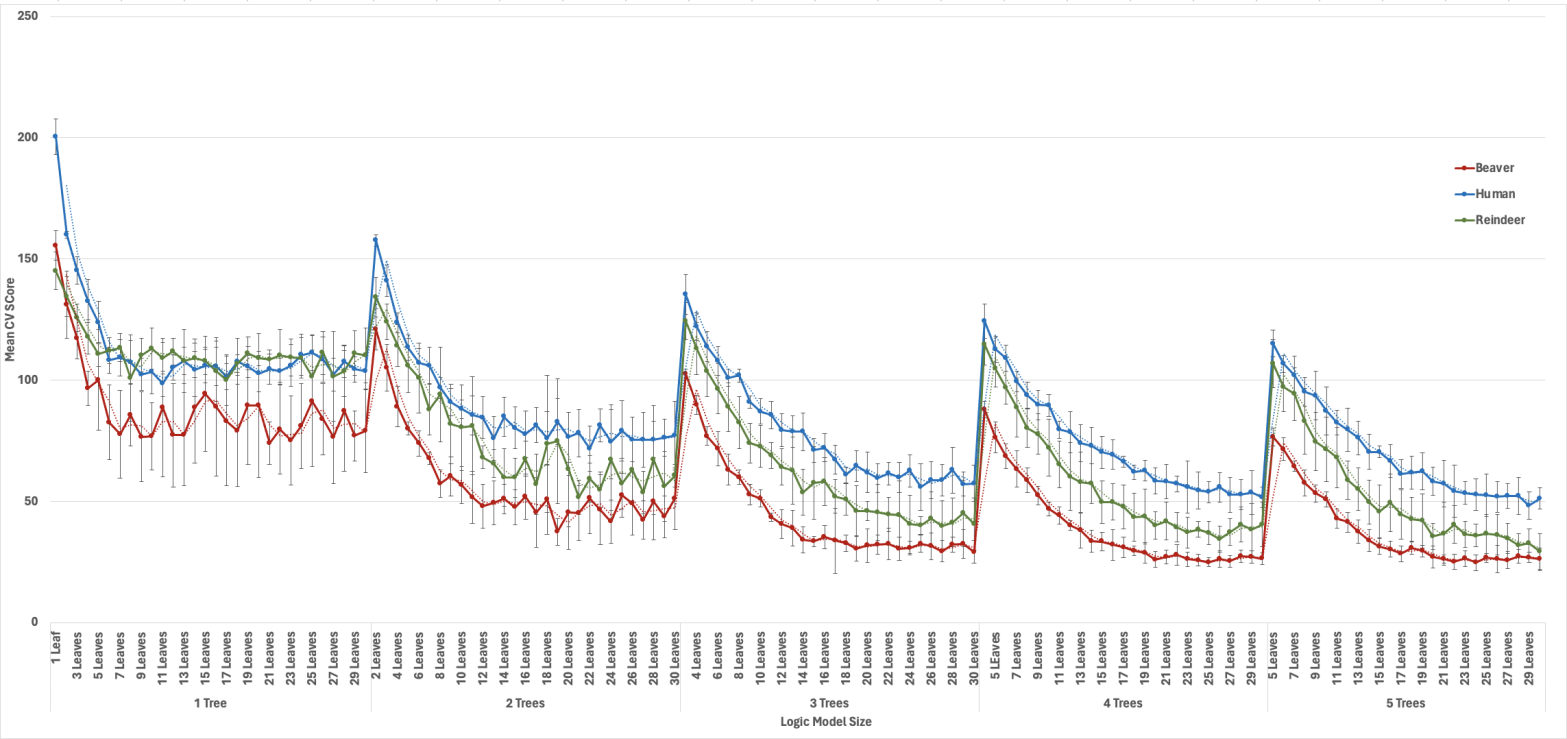
Average cross-validation scores by model size for the determination of the optimal model size parameters for beaver, human, and reindeer-specific logic models. Host-informative logic models were generated for beaver, human and reindeer *E. coli* strains collected from Sweden and Canada using the sequence variation contained within a concatenated sequence consisting of the *asnS–ompF* and *csgDEFG–csgBAC* intergenic regions. To determine the optimal model size for each host group, five independent iterations of logic regression model building were performed to determine the average performance, measured through cross-validation scores, for each model size between 1 and 5 trees and 1 to 30 leaves. the optimal size range was then used to inform the model building portion of the classification analysis for the source attribution of unknown environmental water *E. coli* isolates.

The previous analyses highlight the potential of logic regression for identifying source-informative biomarkers using ITGR sequence data across the *E. coli* genome. *In silico* analyses alone, however, do not necessarily demonstrate the applicability of logic regression for source tracking efforts. To validate our biomarker discovery approach and evaluate its practicality for source attribution analyses, an additional analysis was performed to identify host-informative biomarkers using human-, beaver-, and reindeer-derived *E. coli* isolates that could then be applied to determine the original host source of strains recovered from environmental water samples in Northwestern Sweden. For the sequence selection, the *asnS–ompF* and *csgDEFG–csgBAC* loci were chosen for analysis as they have been previously shown to be particularly source-informative across a wide range of host- and niche-derived isolates (25, 26). To refine the model building process, the “best” size for the models generated from the concatenated *asnS–ompF* and *csgDEFG–csgBAC* sequence was first determined for each of the human, beaver, and reindeer groups. Briefly, five independent iterations of model building were performed for each host group using the concatenated target sequence, with the models ranging in size from 1 to 5 trees and 1 to 30 leaves. Across all model sizes, the beaver models consistently exhibited the lowest average CV-scores, followed by the reindeer and human models (Fig. 2. Interestingly, regardless of their relative performance, the CV-scores for each host group appeared to plateau from 18 leaves onward when the number of trees was set between 3 and 5. As such, for all subsequent model building with the given host range (i.e., beaver, human, and reindeer) and input sequence (i.e., *asnS–ompF* concatenated with *csgDEFG–csgBAC*), the size parameters were restricted to 3 to 4 trees and 18 to 25 leaves.

**Fig 3:**
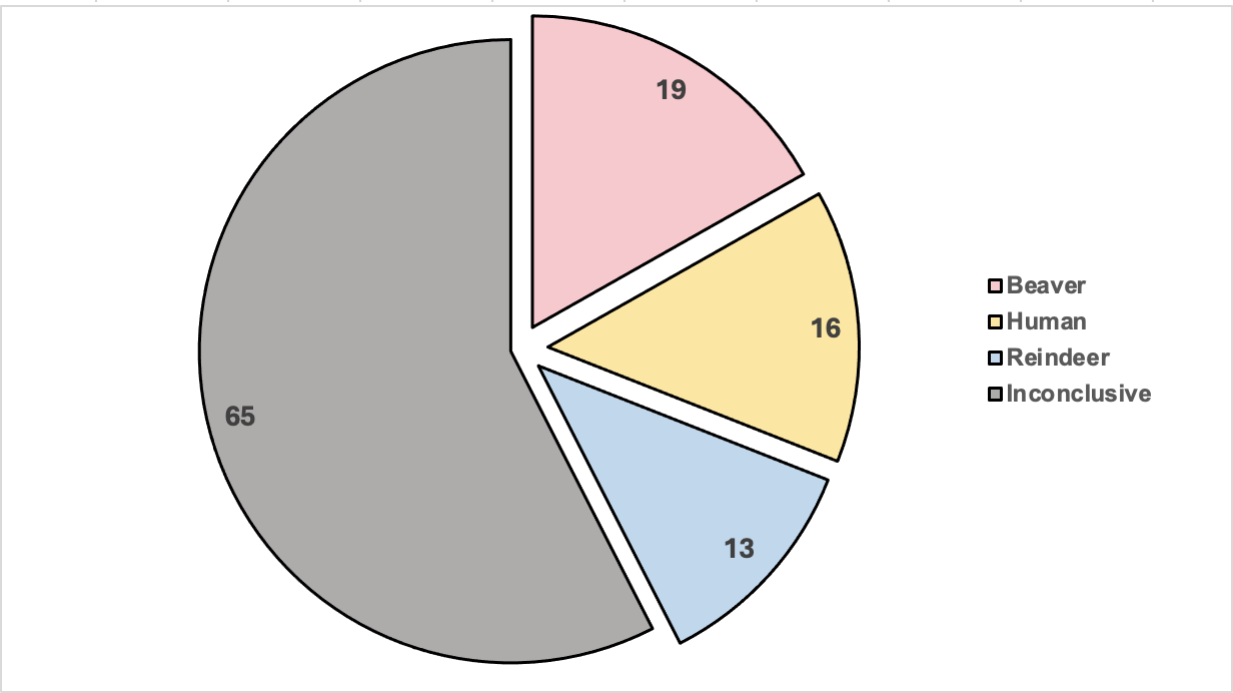
Classification of unknown environmental water *E. coli* isolates according to presumptive original host source based on logic regression analyses. A total of 113 *E. coli* isolates recovered from water samples collected from the Jämtland County region of Northwestern Sweden were classified according to the application of beaver-, human-, and reindeer-specific biomarkers identified through logic regression. Each water isolate was individually classified as being beaver (red), human (yellow), or reindeer (blue) in origin across 273 independent iterations of logic regression analysis. Isolates with classifications reaching at least 80% consensus across the 273 iterations were given a final designation according to their predicted host source, whereas isolates lacking this level of consistency in their classifications or those that were classified into multiple host groups were given an inconclusive final designation (gray).

Having determined the optimal size range for model building, host-informative logic models were generated for the beaver, human, and reindeer strains. A total of 1071 independent iterations of logic regression were performed to generate host-specific biomarkers for each of the beaver, human, and reindeer groups. Given that the generated logic models were to be used for the source attribution of environmental water *E. coli* isolates, only those iterations of logic regression (i.e., seed numbers) that produced host-informative biomarkers of at least 90% specificity across all host groups were retained for the classification analysis. Following screening, 273 logic regression iterations (i.e., in other words, 273 logic models per host source) passed the screening criteria and were used to classify the original host source for the unknown water *E. coli* strains (Table S6). Given that the classifications for each water isolate may vary across each iteration of logic regression analysis, only isolates with classifications that were at least 80% consistent across all 273 iterations were given a final classification. Overall, 63 water isolates were inconsistently classified across the 273 iterations and were left unclassified (Table S7). Interestingly, for two water isolates, over 97% of their classifications were multi-host (i.e., as “Beaver | Human | Reindeer”), and as such these two isolates were also left unclassified for their final designations. The remaining 48 water isolates were classified with a sufficient level of consensus (i.e., of at least 80% across the 273 iterations, including select isolates with classifications that were 100% consistent), including 19 that were designated as beaver in origin, 16 that were human in origin, and 13 that were classified as reindeer in origin (Fig. 3).

## DISCUSSION

*Escherichia coli* is an incredibly diverse species. Although typically known as a common gut commensal in the gastrointestinal tract of humans and various other vertebrate animal hosts (28), this model bacterium also appears to reside in a variety of non-host natural (29) and man-made environments (30–33). This widespread prevalence has led to the designation of *E. coli* as a host and niche generalist, capable of colonizing and transiting across its many niches; however, evidence suggests that the *E. coli* species may be more accurately described as a species complex (34) composed of several “ecotypes” consisting of distinct groups of strains that have each evolved to become specialized to their respective host or niche (35). Given that each potential niche varies widely in the specific stressors (i.e., competing microbiome, temperature, pH, available nutrients, host immune or environmental stressors, etc.) that a strain must adapt to, different strains will be better adapted to certain niches than others, thereby driving their evolution towards host- and/or niche-specialization. Reflecting this, various *E. coli* strains have been documented to exhibit a high degree of host- and/or niche-specificity, and several genetic determinants, ranging from individual SNPs to entire genomic islands (36), reflective of *E. coli* host- or niche-specificity have been identified (35). Although the presence/absence of these genetic markers could influence the ability of a strain to colonize a given host or niche, differences in the relative fitness of strains across different niches could alternatively be reflected in how well they are able to sense and respond to the specific stressors that are present within a given environment. Indeed, ITGRs contain various promoter and repressor sites that can regulate the expression of the flanking genes, which can in turn influence the ability of a given strain to exploit and adapt to a specific niche environment. As the sequences within ITGRs appear to be under strong purifying selection (37), genetic markers of host- and/or niche-specificity may be better reflected through the sequence variation contained within these regulatory, intergenic regions that constitute the “regulome” of *E. coli* (25, 26, 38).

Reflecting this, using logic regression, source-informative SNP-SNP biomarkers (i.e., logic models) of varying levels of sensitivity and specificity were identified in ITGRs across the *E. coli* genome for various host and non-host sources. Although our focus on SNP-SNP modeling could not account for the potential influence of larger scale genomic events (i.e., recombination and/or insertion/deletion events) on the evolution of host-specificity in the *E. coli* species, informative biomarkers could still be generated across a wide range of host sources. It should be noted, however, that various challenges can be associated with establishing an appropriate reference collection of bacterial isolates for host-informative biomarker discovery and subsequent source attribution purposes. Although efforts were made to ensure that the local repository was representative of the global distribution of *E. coli* (Fig. 1), it should be noted that the final number of sequences analyzed (*n* = 846) may not necessarily capture the total diversity of the species, especially given the hundreds of thousands of *E. coli* sequences that have been sequenced to date. This may be particularly important given that the local repository was constructed with a primary focus on collecting enteric and, where possible, commensal strains from a wider range of host sources than in previous studies (25, 26) in order to model and understand the processes underlying the specificity of *E. coli* strains to the gastrointestinal environments of different host species. Indeed, although the genome sequences of a select number of intestinal pathogenic and extraintestinal pathogenic *E. coli* strains were included in this study, key populations such as the globally disseminated, pathogenic, and multi-drug resistant ST131 *E. coli* may not have been properly represented in the repository. Future analyses should thus focus on improving the representation of these key *E. coli* populations and sequence type lineages (i.e., ST131, ST95, etc.), especially for proper risk evaluation when using logic regression-generated biomarkers for source attribution efforts.

Despite these potential limitations, various host- and niche-informative biomarkers were generated with logic regression. In line with previous studies, certain ITGRs appeared to be more informative than others depending on the specific host source being interrogated (26), and no single ITGR appeared to be informative across all host categories that were evaluated in this analysis (Table S4). Furthermore, the biomarkers produced based on the sequence variation contained within single ITGRs alone also varied extensively depending on the specific host source of interest. Indeed, while biomarkers of only limited sensitivity (i.e., less than 30.00%) were mostly generated for bovine-, cat-, chicken-, dog-, human-, rat- and turkey-derived *E. coli* strains, select single ITGRs appeared to encode for biomarkers of much higher sensitivity for the mouse, pig, and sheep groups (Table 1). For instance, the (*rseD-rpoE-rseABC)–nadB* ITGR appeared to be particularly informative for the pig strains, as logic regression was able to identify a biomarker within this locus that was 30.00% sensitive and 97.99% specific to the pig group. Similarly, for the sheep strains, the *yjjP–(yjjQ-bglJ*) locus encoded a biomarker that was 33.33% sensitive and 98.08% specific to the sheep group, whereas the *uspC–flhDC* locus encoded a biomarker that was 33.33% sensitive and 100% specific. Amongst all the host categories, however, the biomarker discovery process appeared to be most effective for generating host-informative biomarkers for mouse-derived *E. coli* strains, as mouse-informative biomarkers exhibiting sensitivity values ranging between 33.33% and 66.67% and specificities from 97.26% to 100.00% were identified across multiple loci, including the *ompC–rcsDB*, *nanCMS–fimB*, *yedR–yedS,* and *csgDEFG–csgBAC* ITGR regions. Interestingly, despite its inclusion as a negative, non-host control group for the other host categories for biomarker discovery purposes, the wastewater strains were consistently found to encode sensitive (i.e., exceeding 35.00% sensitivity) and highly specific (i.e., exceeding 99.00% specificity) biomarkers according to logic regression analysis. As several previous studies have characterized and distinguished these wastewater *E. coli* populations from other representative *E. coli* strains (32, 33, 39, 40), the discovery of several wastewater-specific biomarkers across multiple intergenic loci in the *E. coli* genome provides further evidence that wastewater-derived *E. coli* strains may be fully adapted to the wastewater niche and are thus no longer host-associating.

Although source-informative biomarkers for each host and niche source included in this analysis could be identified within single ITGRs across the *E. coli* genome, the lack of continuity in the source-informative ITGRs and the variable performance of identified biomarkers limits the use of single-ITGR logic models as reliable genetic markers for source tracking efforts. One strategy to improve the efficacy of the biomarker discovery process is to utilize the sequence variation contained across multiple ITGRs at once. Indeed, previous studies have shown that the performance of produced logic models can be improved by appending multiple ITGR sequences together and generating biomarkers from the resulting concatenated sequences (26). Additionally, narrowing the source range interrogated, thereby reducing the extra “noise” from extraneous host and niche sources in the sequence data analyzed, may also improve the resulting logic models produced. As such, to improve upon the biomarker discovery process and modify its application for practical source tracking purposes, additional logic regression analyses were performed using a reduced host range reflecting the major hosts and zoonotic sources of *E. coli* (i.e., human, bovine, swine, and chicken)*,* with concatenated ITGR sequences to produce host-informative biomarkers of improved performance from a consistent input sequence. Surprisingly, despite the reduced host range the performance of the single-ITGR logic models did not necessarily improve across all the host categories interrogated. Indeed, while the human and bovine single-ITGR biomarkers both exhibited significant improvements in performance (Table 2), the performance parameters of the chicken and pig biomarkers were comparable across the biomarker discovery analyses. Regardless of the performance of their single-ITGR biomarkers, however, several concatenated ITGR sequences were identified that appeared to encode better performing, host-informative biomarkers of significant association for each host source interrogated (Table 3). Interestingly, aside from the *yedR–yedS* locus, which was previously found to be particularly informative for bovine- and human-derived *E. coli* strains (26), several additional ITGR targets were identified as potentially containing host-informative sequence variation including the *emrKY–evgAS*, *ibsB–(mdtABCD-baeSR),* and *ompC–rcsDB* intergenic loci. Remarkably, closer inspection of the genes flanking the intergenic sequences represented in the concatenated targets revealed that certain functions were over-represented amongst the identified ITGR targets. Indeed, aside from the *rcsDB* locus, which acts as a master regulator for capsule biosynthesis (41), most of the flanking genes were involved primarily in antibiotic resistance, including: (i) *yedS,* within the previously identified *yedR–yedS* locus, which appears to mediate resistance against carbapenems (42); (ii) *emrKY,* within the *emrKY–evgAS* locus, which encodes for an efflux system that appears to be activate in response to tetracycline (43); (iii) *evgAS*, within the *emrKY–evgAS* locus, which encodes for a two-component regulatory system that controls the expression of several antibiotic resistance genes in *E. coli* (44); (iv) *mdtABCD* and *baeSR*, within the *ibsB–(mdtABCD-baeSR*) locus, which appears to encode for a multidrug efflux pump and its corresponding two-component regulatory system, respectively (45); and (v) *ompC*, within the *ompC–rcsDB* locus, which encodes for an outer membrane porin implicated in resistance to various antibiotics (46) as well as bile salts required for successful colonization of the mammalian gut (47). Interestingly, these findings seem to mirror previous analyses that have identified ITGRs associated with antibiotic resistance genes as particularly informative for human and bovine *E. coli* strains (26). Considering the rates of antibiotic use, particularly overuse, in clinical (48) and agricultural (49) settings, it appears that control over responses against antibiotic stress may be especially important for *E. coli* strains colonizing human and livestock animal hosts.

Generally, the biomarkers produced in this study, both from single ITGR sequence data and from multiple ITGRs combined to form concatenated sequences, exhibited lower predictive power (i.e., lower sensitivities) than the biomarkers that were generated in previous work exploring the use of logic regression for biomarker discovery purposes (25, 26). Several factors could underlie this discrepancy, including that this analysis utilized an *E. coli* genome sequence repository with a broader range of hosts/niches and geographical isolation sites represented, and that this study improved on the logic regression workflow with an iterative approach to model building and the use of 10-fold cross-validation instead of five-fold cross-validation. Remarkably, despite these differences, our findings still highlight the utility of logic regression for identifying host-informative genetic markers using *E. coli* genome sequence data and, importantly, demonstrate the potential for the application of these biomarkers for source attribution purposes. Indeed, although the classification capacity of the biomarkers generated from concatenated sequence data varied depending on the host source and specific concatenated ITGR sequence target, several biomarkers were found to exhibit classification accuracies exceeding 80.00% (Table 3), which appears to be comparable to the classification rates obtained using other supervised learning methods such as support vector machines, random forests, and neural networks (8, 23, 38). Considering that previous studies have demonstrated that other, unsupervised approaches to genomic sequence analysis (i.e., maximum likelihood phylogenetics), are unable to effectively cluster and assign *E. coli* isolates according to host-source (25, 26), our findings provide further evidence for the utility of supervised learning approaches, and specifically logic regression, for modeling and understanding the evolution of host- and niche-specificity within the *E. coli* species.

Importantly, while other studies have used supervised learning for the source attribution of *E. coli* isolates recovered from various human and animal host species (8, 23, 25, 26), to our knowledge this study represents the first to extend the use of biomarkers discovered with supervised machine learning algorithms for the source attribution of environmental isolates with no known host source. Reflecting this, a collection of human-, beaver- and reindeer-specific biomarkers that were produced based on the sequence variation contained within the *asnS–ompF* and *csgDEFG–csgBAC* intergenic loci were later applied to predict the original host source of environmental *E. coli* isolates recovered from river water samples collected in the Jämtland county of Northwestern Sweden. Remarkably, 48 of the 113 total unknown isolates were successfully classified with a sufficient degree of consensus across 273 independent classification trials, and were determined to be either human-, beaver-, or reindeer-derived (Fig. 3).

**Fig 4:**
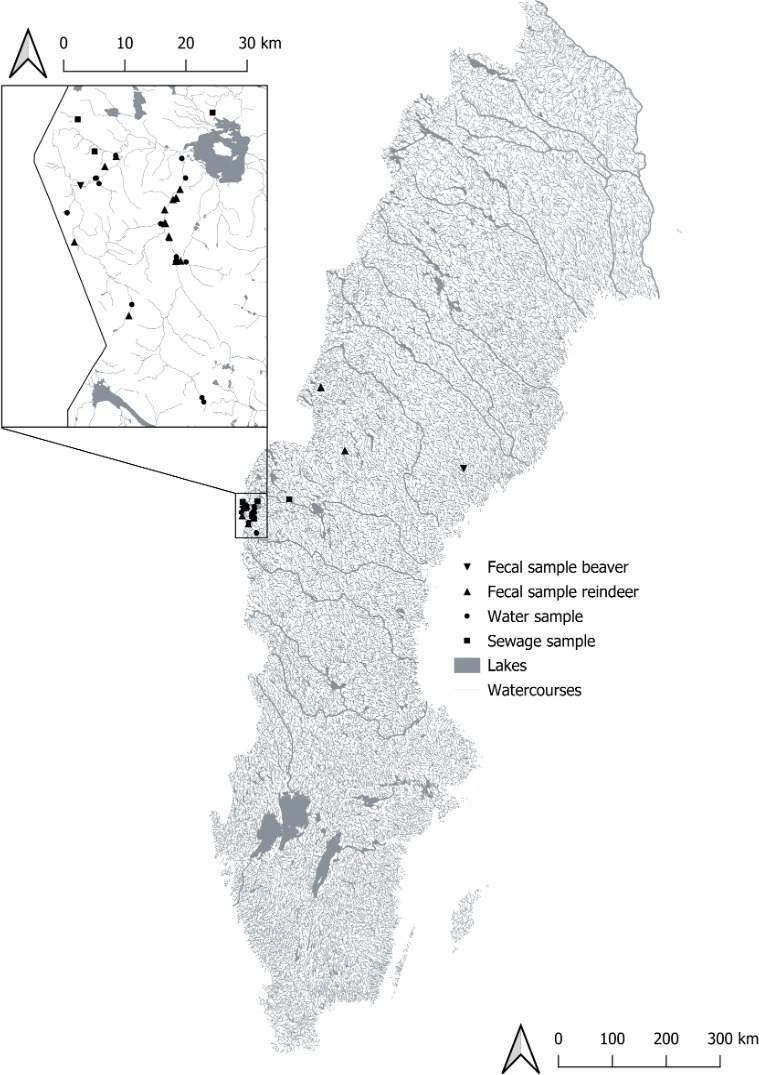
Map of Sweden (© Lantmäteriet) depicting the sampling locations of fecal (*n* = 2 for beaver, *n* = 25 for reindeer), water (*n* = 37) and sewage (*n* = 4) samples. Most samples were taken in the main research area (depicted in the expanded view) located in Jämtland county in Northwestern Sweden, while the remaining samples were taken at other locations (shown on the main map area).

Despite this, 65 environmental water isolates assessed in this study remained unclassified. Aside from two isolates that were given a multi-host designation (i.e., “Beaver | Human | Reindeer”) and thus could represent potential generalist strains (Table S7), the host source classifications for the majority of the water isolates remained undetermined (Fig. 3). Importantly, this significant proportion of unclassified isolates in this analysis points to various areas of improvement for our classification workflow. For instance, 48 environmental isolates were still classified and were given a final host designation with a high degree of consensus based on biomarkers that were produced from only two ITGR sequences. Although the *asnS–ompF* and *csgDEFG–csgBAC* intergenic loci have previously been found to be particularly host-informative (25, 26), several additional potential ITGR targets, including those that were newly identified in this study (Table 3), could have been incorporated into our study to improve upon the classification power of the logic regression workflow. Similarly, the classifications that were made in this analysis were limited to one of three potential host categories based on the presumed predominant sources (i.e., tourists, beavers, roaming reindeer herds, etc.) of fecal *E. coli* isolates impacting the rivers in Northwestern Sweden from which the water samples were taken (Fig. 4). Various other animal sources (i.e., birds, rodents, moose, etc.), however, that could also be introducing fecal contamination to the sampling region, were not represented in this analysis, and *E. coli* isolates derived from these host sources could comprise a considerable proportion of the environmental isolates that were left unclassified in this study. Alternatively, the presence of *E. coli* isolates in the study area could be due to the influence of contamination and/or runoff from the wastewater and sewage treatment infrastructure of the surrounding mountain stations (27). A significant proportion of the unclassified isolates could thus be anthropogenic (i.e., human-derived) in nature; however, these strains might have been left without a final host designation during the classification analysis due to the lack of representation of various important human *E. coli* populations that were not included during the model building process. Finally, while the classification analysis was focused on predicting the host species from which the environmental water isolates could have originated, some of the isolates that were left unclassified could have alternatively belonged to naturalized *E. coli* populations that have become adapted to the natural environment as a primary niche (28, 29). Considering that distinct naturalized populations have been described to reside in river water and sediments (29, 50, 51), some of the river water isolates that were left unclassified in this study could instead represent naturalized strains that have diverged from their host-associated counterparts and were thus not captured by a classification workflow focused on host source attribution. Despite these limitations, the findings presented in this study still highlight the potential of logic regression as a novel approach both for the discovery of host and niche-informative biomarkers in the *E. coli* genome and their practical application for microbial source tracking efforts.

The health and economic burden of waterborne pathogens and their associated diseases (52) calls for methods to track, rapidly and reliably, the sources of fecal contamination in the environment. While a variety of library-dependent and library-independent approaches have been developed to date, thereby contributing to a suite of source tracking methods, microbial source tracking has yet to fully leverage the potential of machine learning for the source attribution of fecally derived microbes detected in the natural environment. Logic regression represents one such approach, as previous work has explored its utility for the detection of genetic biomarkers in *E. coli* that can be predictive of a strain’s original host source (25, 26). Building on these studies, we demonstrate the capability of logic regression for identifying robust host-informative biomarkers within select ITGRs, particularly those flanked by genes mediating functions related to antibiotic resistance, across the *E. coli* genome. Importantly, these discovered biomarkers appear to have practical value for source tracking purposes, as we utilize them for the classification of environmental *E. coli* isolates collected from river water samples collected in the Jämtland County of Northwestern Sweden. While we note some key areas of improvement for our proposed workflow, logic regression appears to be quite effective for biomarker discovery and source attribution purposes and could even represent a novel addition to the microbial source tracking method toolbox.

## MATERIALS AND METHODS

### Bacterial strains for *in silico* whole genome sequence-based biomarker discovery

A local repository of *E. coli* genomes was constructed for biomarker discovery purposes, building on previous *E. coli* genome libraries (26) with a focus on expanding the range of host species represented in the repository. A total of 2925 *E. coli* genome sequences, collected from a range of host species (i.e., bovine, human, pig, sheep, chicken, turkey, mouse, rat, dog, cat, and other animals) and niches (i.e., wastewater), were first downloaded from NCBI and then screened using a set of selection criteria designed to:

1. Maximize the quality of the genome assemblies included in the final library;
2. Remove duplicate genomes and any genomes of strains with mislabeled isolation sources;
3. Minimize the degree of clonal representation amongst genome assemblies recovered from the same sequencing project; and
4. Maximize the temporal (i.e., year of isolation) and geographical (i.e., country of isolation) diversity of strains included in the final library (Fig. 1).

The *E. coli* genome sequences that passed the screening criteria were then used to generate a local repository using BLAST +v2.12.0 (53). All *E. coli* strains used for the biomarker discovery analysis and their relevant metadata can be found in Table S1.

### Selection of *E. coli* intergenic regions for biomarker discovery

Expanding on a set of *E. coli* ITGRs (i.e., *asnS–ompF*, *csgDEFG–csgBAC*, *uspC–flhDC*, *yedS–yedR*) that were previously found to be host-informative (25, 26), 58 additional ITGRs (i.e., 63 total) were selected as candidate loci for the discovery of host source-specific biomarkers via logic regression. Building on previous studies (26), the candidate ITGRs were selected based on the role of the flanking genes in functions that could be associated with host adaptation and colonization (i.e., and therefore potentially host-specificity), including nutrition, adhesion and biofilm formation, colonization factors, antibiotic resistance, and stress resistance (Table S2), as determined after reference to the UniProt (54) and EcoCyc (55) databases. All candidate ITGR sequences were extracted from the genome sequence of the laboratory reference strain *E. coli* K-12 MG1665 with bedtools v2.30.0 (https://github.com/arq5x/bedtools2), and screened against the local repository using BLAST +v2.12.0 (53). Only ITGR sequences that displayed ≥95% coverage with the queried sequence extracted from the reference *E. coli* K-12 MG1665 strain were kept for logic regression analysis. Additionally, ITGR loci that were found to be sparingly represented across the repository (i.e., in less than 750 strains) or those that were either too short (i.e., less than 250 bp in length) or lacked sufficient sequence variation across the strains analyzed (i.e., if over 50% the ITGR sequences extracted from the strains shared over 98% sequence identity) were removed and excluded from downstream analyses. The remaining ITGRs that passed the above screening criteria were then extracted from the strains in the repository with bedtools v.2.30.0 (https://github.com/arq5x/bedtools2), aligned with Clustal Omega (56), and then visualized and refined with the Jalview platform (57). The aligned sequences (available in supplementary information) were then analyzed with logic regression for biomarker discovery purposes.

### Identification of host-informative biomarkers across *E. coli* ITGRs via logic regression

Following previous workflows (25, 26) the sequence variation contained in the candidate ITGRs across each host/niche category in the repository was analyzed using logic regression to identify host source-specific SNP-SNP biomarkers. Specifically, logic regression generates decision trees to predict a binary classification for a given strain, corresponding to whether it originated from a specific host or niche or from some other source of origin. As it uses SNPs as predictive parameters, logic regression can thus generate logic models consisting of SNP-SNP interactions, represented with the Boolean logic terms “AND,” “OR,” and “NOT” that can then serve as biomarkers of host-specificity in *E. coli*, as follows:


$$\mathrm{logit}(E[Y])=\beta_{0}+\beta_{1}L_{1}+\beta_{2}L_{2}+\ldots +\beta_{p}L_{p}$$

Where:

- Y is a binary variable, corresponding to a strain’s membership to one host or niche source group (Y = 1) or some other source of origin (Y = 0).
- β_0_, β_1_, β_2_, …, β_p_ are parameters indicating the degrees of association between the SNP patterns (L) and the prediction outcome (Y).
- L_1_, L_2_, …, L_p_ are the SNP-SNP interactions consisting of Boolean combinations (termed “trees”) of SNP genotypes (termed “leaves”) within the ITGRs.

All logic regression analyses were performed using a custom R script (available in supplementary data). As a massive number of potential models can be built with a varying number of trees of leaves, a simulated annealing algorithm was used with logic regression to select the trees and leaves adaptively based on deviance to find the best fitting model. While previous studies using logic regression limited the model building parameters to 2 trees and 10 leaves to limit the computational burden of the analyses (25, 26), the size of the model is likely to impact the fit of the model produced. Furthermore, the “optimal” model parameters may also vary depending on the specific host source being assessed, and on the specific sequence being analyzed. As such, an iterative model building approach was used in this study, in which logic models of each size ranging from 2 to 3 trees and 15 to 30 leaves were generated and compared with determine the best performing model size for each source category and ITGR. As part of the model building process, the script runs a 10-fold cross-validation and calculates a mean cross-validation test-score (CV-score) to assess the fit for each model. The model size with the lowest mean CV-test score was then selected to be used for downstream logic regression analysis for biomarker discovery purposes, thereby lowering the chances that the models selected will be “overfitted”. Model performance was then evaluated using a “test set” of strains, consisting of 20% of the total number of strains analyzed with logic regression, that was reserved during the model building process. Specifically, all logic models were assessed according to measures of sensitivity and specificity; as described previously (25, 26), sensitivity was defined as the proportion of strains from a target source category that carried a specific SNP pattern, while specificity was defined as the proportion of strains from sources other than the target source category (i.e., all other host or niche sources) that did not carry the SNP biomarker of interest. Finally, a permutation test was performed to assess the validity of each logic model (i.e., host- or niche-specific biomarker) generated by logic regression, and to evaluate the significance of the association between each biomarker and their corresponding source category. To perform the permutation test, the host labels were randomly permuted and the data were re-analyzed with logic regression 1000 separate times. The number of instances where the permuted data sets produced logic models with higher performance values (as measured by the mean of the sensitivity and specificity of the models) than the models produced from the original data were counted, and this value was divided by 1,000 to generate a *P* value.

For the biomarker discovery portion of this study, two separate sets of biomarker discovery trials were run. The first trial included all host/niche source categories in the repository to evaluate the ability of logic regression to identify source-specific biomarkers across an expanded host/niche range, with the “other animal” and wastewater groups serving as negative controls (i.e., strains not associated with any of the target host groups) in the logic regression analysis. To improve on the generated models (i.e., thereby identifying more specific and sensitive biomarkers), a second trial was performed with a reduced host range consisting of only human, bovine, chicken, and pig strains, and with concatenated ITGR sequences as input for logic regression. In addition to sensitivity and specificity measures, biomarker performance was also evaluated using an “accuracy” metric, which was calculated based on the proportion of correct classifications (i.e., including “true positives” for strains derived from a host source that carried the corresponding host-specific biomarker, and “true negatives” for strains derived from other host sources that did not harbor a given host-specific biomarker of interest) that were made according to the test set.

### Bacterial strains for *in vitro* biomarker discovery and application for microbial source tracking of fecal contamination in rivers in Northwestern Sweden

To validate the logic regression-based, biomarker discovery approach, an additional logic regression analysis was performed on physical *E. coli* isolates within the laboratory. A total of 32 fecal samples were collected from beavers and reindeer from 27 sampling sites within the Jämtland county in Northwestern Sweden (Fig. 4). One gram of each fecal sample was diluted in 100 mL of Peptone water (Oxoid, LP0037), plated on Membrane fecal Coliform Agar (mFC, DifcoTM mFC agar, BD Biosciences, 267720) with 0.01% Rosolic acid (DifcoTM Rosolic acid, BD Biosciences, 232281), and then incubated at 44 ± 0.5°C for 22 ± 2 hours. Clearly morphologically distinct blue colonies that grew on the mFC plates were then picked and grown in Lactose Tryptone Lauryl Sulphate Broth (LTLSB, Oxoid, CM0921) supplemented with 4-methylumbelliferyl-β-D-glucuronide (MUG supplement, Oxoid, BR0071E) after incubation for 21 ± 3 hours at 44 ± 0.5°C for the isolation of putative *E. coli* isolates. Confirmed *E. coli* isolates were then stored at −18°C in Brain Heart Infusion Broth (BHI, Oxoid, CM1135) supplemented with 20% glycerol (Apl, 33868). Additional Canadian isolates collected in previous analyses (26) were also provided to supplement the library of *E. coli* strains collected from the animal fecal samples in Sweden. In total, 227 *E. coli* strains were used for the *in vitro* portion of this study, including 51 reindeer (*Rangifer tarandus*) isolates collected from reindeer herds in Jämtland; 44 total beaver isolates, including four collected from local Eurasian beavers (*Castor fiber*) in Sweden and 40 isolates used in previous analyses (25) collected from North American (*Castor canadensis*) beaver populations in Canada; and 133 total human isolates, including 115 isolates recovered from clinical fecal swabs collected at the Alberta Provincial Laboratory for Public Health (ProvLab) for routine microbiological testing (adhering to all ethics requirements; File #: Pro00005478_CLS3 at the University of Alberta) and 26 *E. coli* genome sequences with a global distribution screened from NCBI to bolster the logic regression model building process.

In addition to these fecal isolates, 113 *E. coli* strains were also recovered from environmental water samples collected from mountain creeks feeding into Lake Ånnsjön in the Jämtland County region of Northwestern Sweden (Fig. 4). As the host source of these water *E. coli* strains were unknown, these isolates were used to evaluate the applicability of the logic regression methodology for source tracking purposes (i.e., to classify unknown isolates according to their original host- or niche-source). All information related to the strains used for the targeted *in vitro* logic regression analysis can be found in Table S3.

### *In vitro* validation of logic regression analyses and their application for microbial source tracking

For the *in vitro* logic regression analysis, the *asnS–ompF* and *csgDEFG–csgBAC* ITGRs were chosen as candidate targets as they have previously been shown to be particularly host- and niche-source informative (25, 26, 32). The target ITGRs were amplified in each of the beaver, human, reindeer, and water isolates with PCR using the primers listed in Table 4.

**TABLE 4:** PCR primers used for *in vitro*, targeted ITGR biomarker discovery approach

ITGR target	Primer	Primer sequence (5'–3')	Reference
*asnS–ompF*	*ompF-F*	TACGTGATGTGATTCCGTTC	(58)
	*ompF-R*	TGTTATAGATTTCTGCAGCG	
*csgDEFG–csgBAC*	*csgD-F*	GGACTTCATTAAACATGATG	(58)
	*csgD-R*	TGTTTTTCATGCTGTCAC	

The PCR conditions for the *asnS–ompF* and *csgBAC–csgDEFG* ITGRs were as follows: initial denaturation at 95°C for 4 min, 33 cycles of 95°C for 30 s, 58°C for 30 s, and 72°C for 1 min, followed by a 7 min extension at 72°C. The total volume of each PCR reaction was 50 µL and contained 10 µL of DNA template, 2U KAPA2G Robust Standard DNA Polymerase (Roche, KK5005) and each primer at a concentration of 500 nM. The PCR products were then sequenced bidirectionally with Sanger sequencing by Macrogen Europe (Amsterdam, The Netherlands), concatenated, and then aligned with Clustal Omega (56). The aligned sequences were then manually edited to trim the 3’ and 5’ ends to remove any missing data.

Following a similar approach to the *in silico* analysis, logic regression was used to analyze the sequence variation within the *asnS–ompF* and *csgDEFG–csgBAC* intergenic sequences to identify host-informative biomarkers for the beaver, human, and reindeer isolates. Given that the results from the *in vitro* analysis were to be used to classify the unknown water isolates, an additional step was taken to identify the “optimal” model size parameters used for model building. Using the same custom R script, five random seed numbers were generated to run five separate iterations of model building for each of the beaver, human, and reindeer isolates, with models ranging in size from 1 to 5 trees and up to 30 leaves. The generated CV scores were then plotted against each model size for each host category to identify the “optimal” model sizes for the logic model building process.

After training and optimization, the generated host models were used to attempt to classify the environmental water strains. As the original fecal contributing source of the water strains is unknown, several rounds of classification were performed and the overall results were combined to determine the final classifications of the water isolates. Briefly, 1100 random seeds were generated with R, of which 1071 remained after removing duplicate seed numbers—for each, one iteration of logic regression was performed to produce host-specific logic models for the beaver, human, and reindeer strains. Only those logic building iterations (i.e., seed numbers) that generated logic models that were at least 90% specific across all host categories were selected to be used to classify the water isolates.

Using another custom R script (available in supplementary information), the *asnS–ompF* and *csgDEFG–csgBAC* sequences extracted from the water isolates were then compared with the beaver-specific, human-specific, and reindeer-specific logic models generated for each “iteration” (i.e., seed number) that passed the above criteria. Briefly, a maximum likelihood value was calculated for each water isolate corresponding to the likelihood that it could classified into the beaver, human, and/or reindeer groups, based on whether each isolate’s sequences contained key SNPs associated with each host-specific biomarker. Isolates that received a positive classification (i.e., corresponding to a likelihood value of at least 0.5) against only one host model were tentatively classified as originating from that host source (i.e., water isolates with a positive classification when compared with the human model were tentatively called as being human in origin), whereas isolates that were not classified by any of the host models were left as unclassified. In the case that a water isolate received positive predictions across multiple host models, a comparative evaluation step was used to resolve the classification between the models. Specifically, an evaluation value was calculated for these indeterminately classified isolates, which combined the model specificity value with the prediction/likelihood value assigned to the strain (i.e., essentially reflecting the ability of the model to predict the host source of a given isolate and the confidence in the model’s prediction). The evaluation values corresponding to each host model assigned to the indeterminately classified isolates were then compared—if the difference between these values was greater than 0.2, the isolate was classified according to the host model with the highest evaluation value; conversely, if the difference was less than 0.2, the isolate was given a joint classification between the corresponding host models (i.e., as a potential host generalist *E. coli* isolate). To make a final classification for each water isolate, the “tentative” classifications in each iteration (i.e., seed number) of logic regression that passed the above criteria were combined. Classifications across the iterations remaining that were at least 80% consistent for each isolate were retained as the final classification, and these isolates were assigned to the corresponding host source. Conversely, water isolates with classifications that lacked this level of consistency across iterations or were consistently given an indeterminate (i.e., multi-host) classification were designated as unclassified, with no known host source.

## Data Availability

All relevant data required to evaluate the conclusions presented are included in this article and its supplemental material or will be made available upon request.

## SUPPLEMENTAL MATERIAL

Supplemental sequences aem.00227-24-s0001.txt Aligned sequences used as input for logic regression to classify the water isolates.

Supplemental code aem.00227-24-s0002.txt R code used to perform all the logic regression analyses in this study.

Supplemental tables aem.00227-24-s0003.xlsx Tables S1 to S7.

ASM does not own the copyrights to Supplemental Material that may be linked to, or accessed through, an article. The authors have granted ASM a non-exclusive, world-wide license to publish the Supplemental Material files. Please contact the corresponding author directly for reuse.
